# Cordycepin Induces Apoptosis and Inhibits Proliferation of Human Lung Cancer Cell Line H1975 via Inhibiting the Phosphorylation of EGFR

**DOI:** 10.3390/molecules21101267

**Published:** 2016-09-27

**Authors:** Zheng Wang, Xue Wu, Yan-Ni Liang, Li Wang, Zhong-Xing Song, Jian-Li Liu, Zhi-Shu Tang

**Affiliations:** 1 Shaanxi Collaborative Innovation Center of Chinese Medicinal Resources Industrialization, Shaanxi Province Key Laboratory of New Drugs and Chinese Medicine Foundation Research, Shaanxi Rheumatism and Tumor Center of TCM Engineering Technology Research, Shaanxi University of Chinese Medicine, Xian Yang 712083, China; aiziji_2005@126.com (Y.-N.L.); 1862927248@163.com (L.W.); szx74816@sina.com (Z.-X.S.); 2 Department of Burns and Cutaneous Surgery, Xijing Hospital, Xi’an 710032, China; Zhengshangxuew@163.com; 3 Key Laboratory of Resource Biology and Biotechnology in Western China, Ministry of Education, College of Life Science, Northwest University, Xi’an 710069, China; jlliu@nwu.edu.cn

**Keywords:** cordycepin, H1975 cell line, apoptosis, proliferation, EGFR

## Abstract

Cordycepin is an active component of the traditional Chinese medicine *Cordyceps sinensis* and *Cordyceps militaris* with notable anticancer activity. Though the prominent inhibitory activity was reported in different kinds of cancer cell lines, the concrete mechanisms remain elusive. It was reported that cordycepin could be converted into tri-phosphates in vivo to confuse a number of enzymes and interfere the normal cell function. For the inhibitory mechanism of EGFR inhibitors and the structure similarity of ATP and tri-phosphated cordycepin, human lung cancer cell line H1975 was employed to investigate the inhibitory effect of cordycepin. The results showed that cordycepin could inhibit cell proliferation and induce apoptosis in a dose-dependent manner. Cell cycle analysis revealed that H1975 cells could be arrested at the G_0_/G_1_ phase after cordycepin treatment. The expression levels of apoptosis-related protein Caspase-3 and Bcl-2 and phosphorylated expression levels of EGFR, AKT and ERK1/2 were all decreased compared with the control group stimulated with EGF. However, the protein expression levels of proapoptotic protein Bax and cleaved caspase-3 were increased. These results implied that cordycepin could inhibit cell proliferation and induce apoptosis via the EGFR signaling pathway. Our results indicated that there was potential to seek a novel EGFR inhibitor from cordycepin and its chemical derivatives.

## 1. Introduction

Lung cancer remains one of the most common types of cancer worldwide. In addition, non-small cell lung cancer (NSCLC) accounts for approximately 85% of all lung cancers, with a five-year survival rate in advanced NSCLC of less than 5% [1]. Epidermal growth factor receptor (EGFR), over-expressing in a large number of human tumors, is a member of the erbB family which includes erbB2/HER2, erbB3/HER3 and erbB4/HER4 [2,3]. The binding of ligands to the extracellular domain of the EGFR activates the receptor tyrosine kinase and triggers downstream signaling pathways to regulate proliferation and apoptosis of cancer cells. Therefore, EGFR and its signal transduction pathway have undergone the most advanced research as a therapeutic target and a series of EGFR inhibitors have been designed, synthesized, and some of them have been used in clinical trials, such as gefitinib and erlotinib [4,5]. These EGFR inhibitors can bind to the kinase domain to impede the binding of ATP to prevent phosphorylation of EGFR, resulting in the interruption of the signal transduction. However, acquired resistance to these inhibitors frequently develops after a median of nine to 13 months, mainly for the EGFR mutation [6,7,8,9]. Furthermore, the EGFR T790M mutation is present in approximately 50% to 60% of resistant cases [10]. Though the third generation of EGFR inhibitors, such as WZ-4002 [11] and CO-1686 [12], has been developed to conquer this intractable problem, the drug resistance has been improved substantially but not completely solved. Therefore, it is imperative to seek novel EGFR inhibitors to overcome the drug resistance.

Traditional Chinese medicine is a treasure trove gifted by nature to discover novel compounds. Cordycepin, 3’-deoxyadenosine (9-3-deoxy-β-d-ribofuranosyl) adenine), is an active ingredient that was isolated from *Cordyceps militaris* in 1951, which exhibits a variety of clinical health effects including immunomodulatory, anticancer, antioxidant, anti-inflammatory and anti-microbial activities [13,14,15]. It was reported first that cordycepin could inhibit the biosynthesis of purine and participate in the synthesis of RNA and/or DNA to exert its biological activity [16]. With the development of cell biology and molecular biology, studies about cordycepin regulating cancer cell progress appear more and more frequently. Choi and co-workers [17] reported that cordycepin could induce human leukemia cell apoptosis through a signal involving a ROS (Reactive Oxygen Species) mediated caspase pathway. Kazuki Nakamura [18] proved that the antitumor effect of cordycepin on lung carcinoma cells was induced via the stimulation of the adenosine A_3_ receptor. Liou et al. [19] reported that cordycepin could suppress integrin/FAK signaling and the epithelial-mesenchymal transition in hepatocellular carcinoma. Lee and co-workers [20] suggested that cordycepin could inhibit the migration and invasion of LNCap human prostate carcinoma cells by inactivation of AKT, resulting in the down-regulation of the TJs (Tight Junctions) and MMPs (Matix Metalloproteinases). Chen and co-workers [21] investigated the effect of cordycepin on C6 glioma cells and showed that cordycepin could induce the apoptosis of C6 glioma cells via the adenosine 2A receptor-p53-caspase-7-PARP pathway. Though almost all the studies reported that cordycepin could inhibit cell proliferation and induce apoptosis, the concrete mechanisms were not identical.

**Figure 1:**
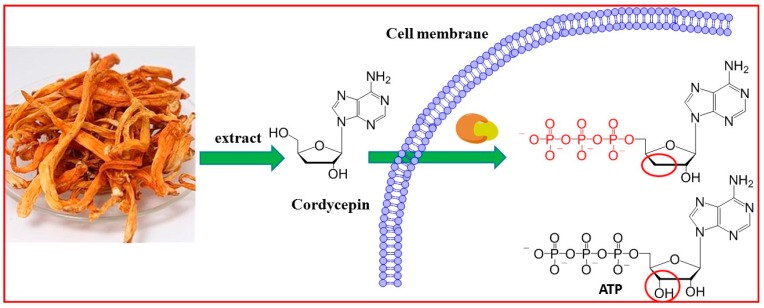
Chemical structure of cordycepin and ATP.

It is confirmed that cordycepin can be converted into 5’ mono-, di- and tri-phosphates in vivo to inhibit the activity of enzymes and interfere a number of biochemical and molecular processes [22,23]. The role of EGFR inhibitors is exerted by competitively fitting to the ATP binding pocket of EGFR to prevent the signal transduction [24]. The chemical structure of tri-phosphated cordycepin is very similar to ATP. Therefore, it is predicted that EGFR may be confused by cordycepin through incorporating it in the ATP binding pocket to affect the normal function of EGFR (Figure 1). For the similarity of tri-phosphated cordycepin with ATP, it was predicted that cordycepin may confuse EGFR for the incorporation of cordycepin in the ATP pocket to affect the normal function of EGFR (Figure 1).

Taken together, we hypothesize that cordycepin can interrupt EGFR signal transduction to inhibit lung cancer cell proliferation and induce apoptosis. In the present study, we extracted and purified cordycepin form *Cordyceps militaris* and explored the anticancer effect and its mechanisms. Human lung cancer cell line H1975 was employed and the phosphorylation of EGFR and its downstream molecular proteins AKT and ERK1/2 were investigated to verify the inhibitory role of cordycepin.

## 2. Results and Discussion

### 2.1. Cordycepin Extraction and Purification

To get enough cordycepin, *Cordyceps militaris* was extracted in water and the extraction solution was purified by Amberlite 732 column chromatography (0.15 mol/L ammonia water) and recrystallization in H_2_O. The chemical structure of the isolated cordycepin was determined by ^1^H-NMR, ^13^C-NMR and MS. ^1^H-NMR (400 MHz, DMSO): δ 8.39 (s, 1H), 8.17 (s, 1H), 7.32 (s, 2H), 5.85 (d, *J* = 2.5 Hz, 1H), 5.69 (d, *J* = 4.0 Hz, 1H ), 5.25 (t, *J* = 5.7 Hz, 1H), 4.62 (m, 1H), 4.38 (m, 1H ), 3.57–3.63 (m. 2H), 1.83–1.95 (m, 2H); ^13^C-NMR (400 MHz, DMSO): δ 147.53, 151.59, 156.33, 138.91, 118.95, 90.95 , 81.01, 74.69, 62.98, 33.92. ESI-MS for C_10_H_1__3_N_5_O_3_ [M + H]^+^: calcd: 251.12, found: 252.12, mp 229~230 °C.

### 2.2. Cordycepin Inhibits H1975 Cell Proliferation

**Figure 2:**
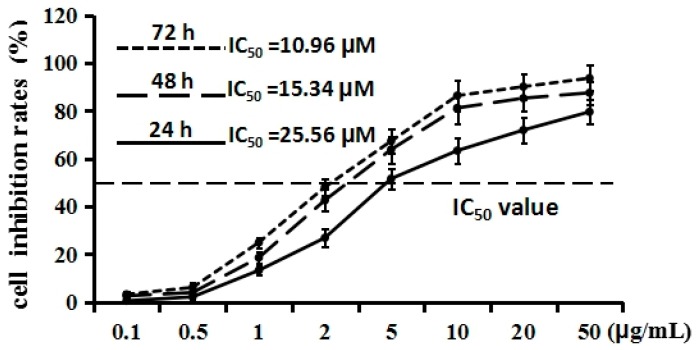
Effects of cordycepin on the cell proliferation of H1975 cells in vitro. H1975 cells were treated with various concentrations (0.1, 0.5, 1, 2, 5, 10, 20, 50 µg/mL) of cordycepin for 24, 48 and 72 h, respectively. Error bars represent means ± SEM of *n* = 3.

The effect of cordycepin on proliferation of H1975 cells was determined by MTT assay. As shown in Figure 2, after treatment with various concentrations (0.1, 0.5, 1, 2, 5, 10, 20, 50 µg/mL) of cordycepin for 24, 48, and 72 h, the proliferation of H1975 cells was inhibited moderately. The line chart exhibited that cordycepin could inhibit the proliferation of the H1975 cells’ concentration dependently. Half-maximal inhibitory concentration (IC_50_) values were calculated by SPSS. The IC_50_ value of 48 h (15.34 µM) was higher than that of 72 h (10.96 µM), but was lower than the IC_50_ value of 24 h (25.56 µM). These results indicated that cordycepin could inhibit the proliferation of H1975 cells in a dose-dependent manner. To elucidate the inhibitor potency of cordycepin, gefitinib, from the first generation of EGFR inhibitors, was employed as a positive control. After treatment with various concentrations (0.1, 0.5, 1, 2, 5, 10, 20, 50 µg/mL) of gefitinib for 48 h, the proliferation of H1975 cells was inhibited prominently. The IC_50_ value was 2.34 µM, dramatically lower than that of cordycepin after 48 h of treatment. These results implied that cordycepin could inhibit the proliferation of H1975, but the inhibitor potency was notably lower than that of gefitinib, indicating that chemical structure modification may be a good choice to enhance the inhibitor potency of cordycepin to seek more potent EGFR inhibitors.

### 2.3. Cordycepin Induces G_0_/G_1_ Phase Arrest in H1975 Cells

**Figure 3:**
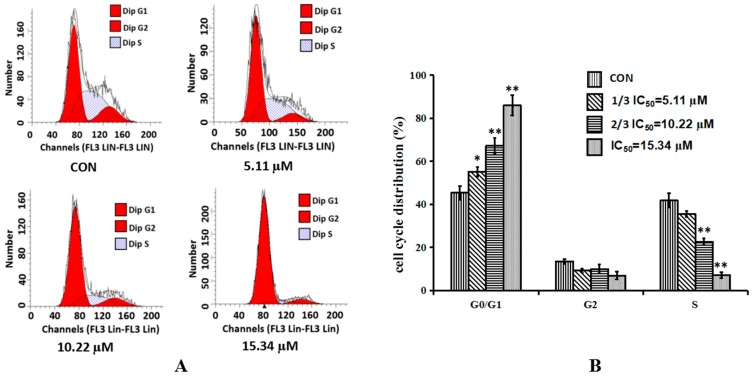
Effects of cordycepin on the cell cycle of H1975 cells in vitro. (**A**) H1975 cells were treated with cordycepin at 5.11 µM, 10.22 µM and 15.34 µM for 48 h and the DNA content was analyzed by flow cytometry; (**B**) Histogram summarized the results of (**A**). Cell numbers at G_0_/G_1_, G_2_ and S phases were counted and the percentage was calculated. Error bars represent means ± SEM of *n* = 3. * *p* < 0.05 and ** *p* < 0.01 vs. control group.

To further verify the effect of cordycepin on cell proliferation, the cell cycle distribution was analyzed by flow cytometry. As shown in Figure 3, cordycepin significantly increased the number of cells in the G_0_/G_1_ phase in a dose-dependent manner. Contrarily, the number of cells in the S phase was decreased along with the increase of the treatment concentration. It was concluded that cordycepin could arrest the cell cycle of H1975 at the G_0_/G_1_ phase and the effect of cordycepin was shown to have a dose-dependent manner.

### 2.4. Cordycepin Induces Apoptosis and Regulates Apoptosis-Related Proteins Bcl-2 and Caspase-3 in H1975 Cells

**Figure 4:**
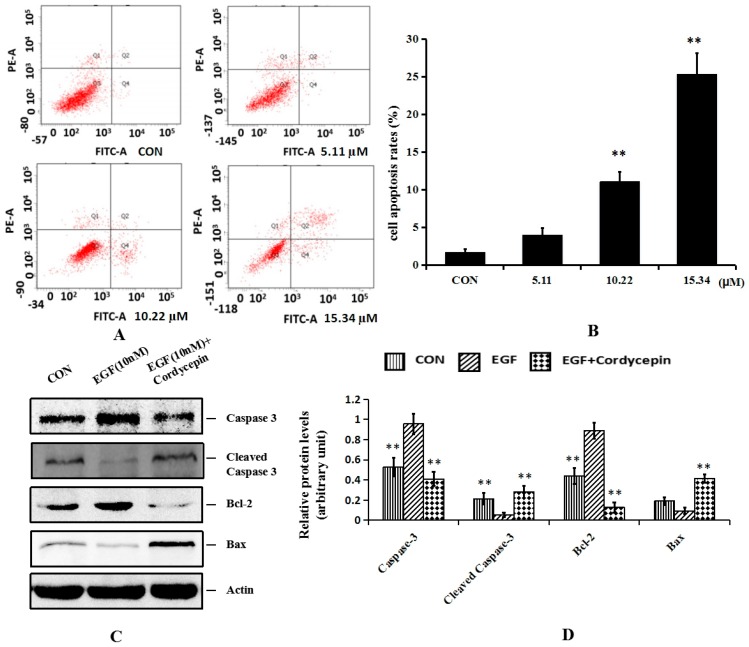
Effects of cordycepin on the apoptosis of H1975 cells and related protein caspase-3 and Bcl-2 in vitro. (**A**) H1975 cells were treated with cordycepin at 5.11 µM, 10.22 µM and 15.34 µM for 48 h and cell apoptosis was analyzed by flow cytometry; (**B**) Histogram summarized the results of (**A**). The activity of H1975 was evaluated by Annexin-V/PI staining; (**C**) Western blot showed the protein level of caspase-3, cleaved caspase-3, Bax and Bcl-2 following the cordycepin (10.22 µM) treatment for 24 h. Actin served as an equal loading control; (**D**) Histogram summarized the results of (**C**). Error bars represent means ± SEM of *n* = 3. ** *p* < 0.01 vs. control group.

Flow cytometry was also carried out to analyze the apoptosis rate of H1975 cells after treatment with cordycepin in the same concentration with cell cycle distribution analysis. As shown in Figure 4, Annexin V-FITC positively and PI negatively (Q4 quadrant) were considered to represent early apoptotic cells; Annexin V-FITC positively and PI positively (Q2 quadrant) represented late apoptotic cells; and Annexin V-FITC negatively and PI negatively (Q3 quadrant) indicated non-apoptotic cells. Q2 and Q4 represented the apoptotic cells. After treatment with cordycepin for 48 h, the number of Q3 quadrant cells was reduced, whereas the numbers of both early and late apoptotic cells were increased significantly in a dose-dependent manner. The treatment with 15.34 µM cordycepin induced early and late apoptosis in approximately 24.3% ± 1.56%.

Apoptosis is a complex, multistage process involving many genes. There are three known ways to induce apoptosis: the death receptor pathway, the mitochondrial pathway and endoplasmic reticulum stress–induced apoptosis. The mitochondrial pathway is the one which has been relatively well studied and understood and is controlled mainly by members of caspase-3, cleaved caspase-3, Bax and Bcl-2. In the present study, the protein expression levels of caspase-3, cleaved caspase-3, Bax and Bcl-2 were detected with treatment of two-thirds IC_50_ (10.22 µM) of MTT assay. Casepase-3 is a type of proteinase which has a central role in the execution phase of cell apoptosis only when it has been activated. Cleaved caspase-3 is the active form of caspase-3 which undergoes proteolytic processing at conserved aspartic residues to produce two subunits, large and small, that dimerize to form the active enzyme. Bax and Bcl-2 are two members of the Bcl-2 family. Bax is necessary for mitochondrial outer membrane permeabilization and it can be inhibited by the antiapoptotic protein Bcl-2. As shown in Figure 4C,D, group 1 was the control. Groups 2 and 3 were stimulated with 10 nM of EGF. The results revealed that following cordycepin treatment, the protein expression levels of caspase-3 and Bcl-2 were all decreased, while the protein expression levels of cleaved caspase-3 and proapoptotic protein Bax were all increased. These indicated that down-regulation of caspase-3 and Bcl-2 and up-regulation of cleaved caspase-3 and Bax were associated with the apoptotic effect of cordycepin on H1975 cells. These results were consistent with the previous study where cordycepin induced apoptosis via down-regulating the protein level of caspase-3 and Bcl-2 [25].

### 2.5. Cordycepin Inhibits the Phosphorylation of EGFR, AKT and ERK1/2 in H1975 Cells

**Figure 5:**
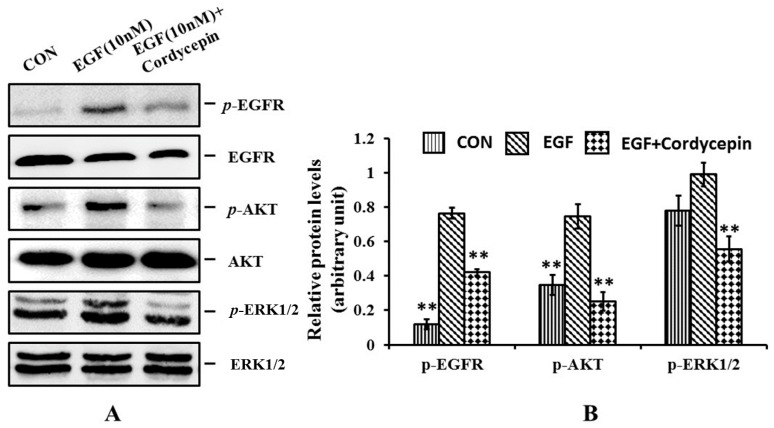
Effects of cordycepin on the apoptosis of H1975 cells and related proteins caspase-3 and Bcl-2 in vitro. (**A**) Western blot showed the phosphorylation level of caspase-3 and Bcl-2 following the cordycepin (10.22 µM) treatment for 24 h; (**B**) Histogram summarized the results of (**A**). Error bars represent means ± SEM of *n* = 3. ** *p* < 0.01 vs. control group.

Though there are many studies on the anticancer activity of cordycepin, the exact mechanism is still elusive. In this study, the phosphorylation level of EGFR and its downstream signal proteins AKT and ERK1/2 were determined by Western blot assay. As shown in Figure 5, group 1 was the control. Groups 2 and 3 were stimulated with 10 nM of EGF to enhance the expression level of the phosphorylated protein. Group 3 was treated with two-thirds IC_50_ (10.22 µM) of cordycepin. The results showed that the expression levels of phosphorylated EGFR, AKT and ERK1/2 were all increased after the EGF stimulation, indicating that the addition of EGF could enhance the phosphorylated protein levels. The columns of group 3 treated with cordycepin were all notably lower than those of group 2. These results implied that cordycepin could inhibit the phosphorylation of EGFR, AKT and ERK1/2. Taken together, it was concluded that cordycepin could inhibit EGFR phosphorylation and its downstream signaling pathway.

## 3. Experimental Section

### 3.1. Cordycepin Extraction and Purification

*Cordyceps militaris* was extracted by water and cordycepin was isolated from the extraction solution by Amberlite 732 column chromatography (Tianjin Bohong Resin Technology Co. Ltd., Tianjin, China) (0.15 mol/L ammonia water) and recrystallization in H_2_O. The purity of the isolated cordycepin was determined by HPLC (Waters, Milford, CT, USA) (Sun Fire™ C_18_, 4.6 mm × 150 mm, 5 μm), using 15% MeOH as mobile phase with a flow rate of 1.0 mL/min. The chemical structure of the isolated cordycepin was identified by ^1^H-NMR (Bruker Avance-400 MHz spectrometer, Billerica, MA, USA), ^13^C-NMR (Bruker Avance-400 MHz spectrometer) and MS (LTQ-KL) (Thermo Fisher, Waltham, MA, USA).

### 3.2. Cell Culture and MTT Assay

Human lung cancer cell line H1975 was purchased from Maoxin Biotechnology Co. Ltd. (Xi’an, China). H1975 cells were pelleted and grown in Dulbecco’s Modified Eagle Medium (DMEM, Gibco, Grand Island, NY, USA) supplemented with 10% fetal calf serum (Gibco),100 U/mL penicillin and 100 U/mL streptomycin, and incubated at 37 °C in a 5% (*v*/*v*) CO_2_ humidified atmosphere for 24 h. Then, H1975 cells were treated with cordycepin at 0.1 µg/mL, 0.5 µg/mL, 1µg /mL, 2 µg/mL, 5 µg/mL, 10 µg/mL, 20 µg/mL and 50 µg/mL for 24 h, 48 h and 72 h, respectively. The medium was removed and the cells were incubated for 4 h in the presence of 5 mg/mL of MTT at 37 °C. The MTT solution was removed and 150 μL of DMSO per well was added. After thorough mixing, absorbance of the wells was read in a SpectraMax 190 Spectrophotometer (Molecular Device, Sunnyvale, CA, USA) at test and at reference wavelengths of 490 nm.

### 3.3. Flow Cytometry Analysis

Cell cycle distribution was analyzed by flow cytometry (Becton, Dickinson and company, Franklin Lakes, NJ, USA). Primary H1975 cells were divided into control, one-third IC_50_ value (5.11 μM), two-thirds IC_50_ value (10.22 μM) and IC_50_ value (15.34 μM) group. Then 48 h after treatment, cells were harvested, rinsed with PBS, fixed with 95% (*v*/*v*) ice-cold ethanol and re-suspended in staining buffer containing FITC-Annexin V and propidium iodide (PI). The mixture was then incubated in the dark at room temperature for 15 min. DNA contents of stained nuclei were analyzed, and the cell number in each cycle phase was calculated.

Cell apoptosis was detected using Annexin V-FITC Apoptosis Detection Kit I (BD Biosciences, San Diego, CA, USA) according to the manufacturer’s instruction. H1975 cells were treated with different concentration of cordycepin for 48 h according to the cell cycle distribution experiment. Cells were then digested with 0.25% trypsin, washed twice with cold PBS, re-suspended in binding buffer and then incubated with Annexin V-FITC and PI for 15 min at room temperature in dark. Samples were then analyzed by FACS Calibur (BD Bioscience). The percentage of stained cells was determined using BD FACSDiva software (Becton, Dickinson and company, Franklin Lakes, NJ, USA).

### 3.4. Western Blot Analysis

H1975 cells were washed with ice-cold phosphate-buffered saline (PBS) and lysed using RIPA buffer supplemented with protease and phosphatase inhibitor mixtures (Heart Biological Technology Co. Ltd., Xi’an, China) on ice. Lysates were separated by centrifugation at 4 °C and 14,000 *g* for 10 min. Protein concentration was determined by BCA assay (Pierce, CO, USA). 50 mg total protein was subjected to sodium dodecyl sulfate-polyacrylamide gel electrophoresis (SDS-PAGE) and transferred to PVDF membranes (Millipore, Bedford, MA, USA). After blocking with 5% non-fat milk, the membranes were incubated with rabbit mAbs specific for p-AKT (CST, #4060), AKT (CST, #4691), p-ERK1/2 (CST, #4370), ERK1/2 (CST, #4695), p-EGFR (CST, #2234), EGFR(CST, #4267s), Bax (ABcam, ab69643), Bcl-2 (Abcam, ab32124), anti-caspase3 (Abcam, ab2171) and anti-cleaved caspase-3 (ABcam, ab32024) overnight at 4 °C, next day the membranes were incubated with horseradish peroxidase conjugated secondary antibodies (1:3000) 37 °C for 1 h. Then immunoreactive proteins were visualized using ECL Western blotting detection reagent (Millipore, Billerica, MA, USA) and detected using MultiImage Light Cabinet Filter Positions (Alpha Innotech, San Leandro, CA, USA).

### 3.5. Statistical Analysis

Each experiment was repeated at least three times, and the data are presented as the mean ± SEM. Statistical differences between groups were analyzed by Student’s *t*-test or the Mann-Whitney *U* test as appropriate using a SPSS 13.0 program. * *p* < 0.05 was considered statistically significant.

## 4. Conclusions

Cordycepin is an active component of *Cordyceps* *sinensis* and *Cordyceps militaris.* Its anticancer activities have been proved in clinical trials. However, cell proliferation, cell cycle progression and apoptosis are very complicated and involve a number of signaling proteins which cooperate to ensure normal cell function. In the present study, the effects of cordycepin on the human lung cancer cell line H1975 were investigated via MTT assay, flow cytometry analysis and Western blot analysis. The results showed that cordycepin could inhibit the cell proliferation with an IC_50_ value of 15.34 μM. Flow cytometry analysis revealed that H1975 cells could be arrested at the G_0_/G_1_ phase and the apoptosis was induced in a dose-dependent manner. The expression levels of apoptosis-related proteins caspase-3 and Bcl-2 were decreased and the proapoptotic protein Bax and cleaved caspase-3 were increased after cordycepin treatment. Western blot analysis displayed that the phosphorylation levels of EGFR, AKT and ERK1/2 were all decreased after cordycepin treatment. AKT and ERK1/2 are the key protein kinases of the EGFR downstream signaling pathway. These results implied that the inhibitory role of cordycepin on H1975 may be exerted through down-regulation of the phosphorylation level of EGFR. It was firmly believed that cordycepin exerts its anticancer activity via multiple mechanisms. Though this study just revealed the tip of the iceberg, the inhibition effect of cordycepin on EGFR could provide useful information to modify the chemical structure of cordycepin to seek more potent anticancer agents.
